# Examining fidelity in the INFORM trial: a complex team-based behavioral intervention

**DOI:** 10.1186/s13012-020-01039-2

**Published:** 2020-09-16

**Authors:** Liane R. Ginsburg, Matthias Hoben, Adam Easterbrook, Elizabeth Andersen, Ruth A. Anderson, Lisa Cranley, Holly J. Lanham, Peter G. Norton, Lori E. Weeks, Carole A. Estabrooks

**Affiliations:** 1 School of Health Policy & Management, Faculty of Health, York University, Toronto, Ontario M3J 1P3 Canada; 2 Faculty of Nursing, University of Alberta, Edmonton, Alberta T6G 1C9 Canada; 3 School of Nursing, Thompson Rivers University, Kamloops, British Columbia V2C 0C8 Canada; 4 School of Nursing, University of North Carolina, Chapel Hill, North Carolina 27599-7460 USA; 5 Lawrence S Bloomberg Faculty of Nursing, University of Toronto, Toronto, Ontario M5T 1P8 Canada; 6 University of Texas Health Science Center San Antonio, University of Texas, San Antonio, Texas 78229 USA; 7 Cumming School of Medicine, University of Calgary, Calgary, Alberta T2N 4 N1 Canada; 8 School of Nursing, Faculty of Health, Dalhousie University, Halifax, Nova Scotia B3H 4R2 Canada

**Keywords:** Trial fidelity, Process evaluation, Nursing homes

## Abstract

**Background:**

Fidelity in complex behavioral interventions is underexplored. This study examines the fidelity of the INFORM trial and explores the relationship between fidelity, study arm, and the trial’s primary outcome—care aide involvement in formal team communications about resident care.

**Methods:**

A concurrent process evaluation of implementation fidelity was conducted in 33 nursing homes in Western Canada (Alberta and British Columbia). Study participants were from 106 clinical care units clustered in 33 nursing homes randomized to the Basic and Enhanced-Assisted Feedback arms of the INFORM trial.

**Results:**

Fidelity of the INFORM intervention was moderate to high, with fidelity delivery and receipt higher than fidelity enactment for both study arms. Higher enactment teams experienced a significantly larger improvement in formal team communications between baseline and follow-up than lower enactment teams (*F*(1, 70) = 4.27, *p* = .042).

**Conclusions:**

Overall fidelity enactment was associated with improvements in formal team communications, but the study arm was not. This suggests that the intensity with which an intervention is offered and delivered may be less important than the intensity with which intervention participants *enact the core components of an intervention*. Greater attention to fidelity assessment and publication of fidelity results through studies such as this one is critical to improving the utility of published trials.

Contributions to the literature

- Although comprehensive models of fidelity assessment exist, recent systematic reviews indicate fidelity frameworks are rarely used and fidelity receipt and enactment are poorly reported.
- By providing a comprehensive, theory-based examination of fidelity in a complex, behavioral intervention (the INFORM trial), this study enhances understanding of how health service interventions are implemented and why they succeed or fail.
- Study findings underscore the need for comprehensive fidelity assessment and suggest more attention needs to be paid to how, and how well, intervention participants can *enact* a complex intervention than to the intensity of intervention *delivery*.

## Background

Funders and investigators make substantial investments in interventions designed to improve healthcare delivery or outcomes, yet they often fail [1] or have limited success or declining success when replicated [2]. The theoretical soundness of an intervention can help explain intervention success or failure [3]. Process evaluations are studies that run in parallel to interventions to understand intervention processes. Process evaluations often include the examination of intervention fidelity and can also enhance understanding of why health service interventions succeed or fail [4, 5]. Assessing fidelity (defined as the extent to which an intervention is delivered and implemented as intended or as per protocol [6]^1^) helps to make clear the mechanisms of impact in a trial—knowledge that is crucial for replication [7, 8] and for drawing unequivocal conclusions about an intervention’s effectiveness [9]. Ignoring fidelity increases the risk of accepting ineffective interventions and of discarding robust interventions that are poorly implemented. *Concurrent* rather than *retrospective* process evaluations are needed, including concurrent fidelity studies. Concurrent process evaluations ensure the theory behind the design of interventions is considered when interventions are evaluated [10], and they accurately capture implementation experiences in real time.

In complex interventions, where there may be multiple mechanisms of impact compared to simple interventions, assuring fidelity is challenging [9] and its examination is particularly important. There is, however, little in the published literature regarding fidelity in complex behavioral interventions [6]. The INFORM study [11] (**I**mproving **N**ursing Home Care Through **F**eedback **O**n perfo**RM**ance data) is a complex three-arm behavioral trial designed to increase the involvement of unregulated care aides in formal team communications about resident care in nursing homes [12]. This paper reports on a mixed-methods study that examines trial fidelity in INFORM and explores the relationship between fidelity, study arm, and the trial’s primary outcome—care aide involvement in formal team communications about resident care.

### Intervention fidelity

The effectiveness of interventions depends on providers delivering the intervention as intended and participants actively engaging with the intervention [9]. Fidelity is therefore influenced by all those who are involved in delivering or receiving an intervention. Various conceptual models of fidelity exist [7, 13–16], and key dimensions are articulated by Bellg et al. in the treatment fidelity model [7]. *Fidelity delivery* is about delivering an intervention consistently, as per protocol, and minimizing contamination. *Fidelity receipt* reflects participants’ receipt and understanding of the intervention components and their capacity to use the skills taught. *Fidelity enactment* reflects participants’ actual performance of intervention skills or implementation of the core intervention components in the intended situation. Bellg and colleagues laid out the dimensions of fidelity delivery, receipt, and enactment and suggest a compendium of approaches (including checklists, observation, document analysis, and interviews) suitable for assessing these dimensions. However, recent systematic reviews indicate that fidelity receipt and enactment are generally underexamined and underreported [9, 17] and/or poorly reported [18]. According to one of these reviews, only 20% of studies used a fidelity framework [9]. Fewer than half of the studies in the review measured both fidelity delivery and fidelity receipt and enactment [9]. Comprehensive, theory-based examinations of fidelity in complex, contextualized interventions are needed to advance understanding of trial effectiveness.

As we address fidelity, it is important to acknowledge ongoing debate about the importance of fidelity versus the need for adaptation [19]. Scholars increasingly suggest that interventions designed for dynamic real-world settings need to be contextualized—there is a need “to balance standardization of [intervention] form and content with responsivity to context.” [20] Others point out that interventions are routinely adapted in practice; thus, adaptation is an ecological reality though it is not well understood [19]. While acknowledging the importance of this debate in the broader implementation literature, this paper focuses on a better understanding of processes important to intervention fidelity. We suggest that fidelity (and related adaptations) can be understood by assessing whether *core components* of an intervention are (1) identified based on the intervention’s underlying theory and (2) delivered and implemented as per protocol [21].

### Study objectives

1. To examine the fidelity of the INFORM intervention—in particular, to what extent the core components of INFORM were (a) delivered, (b) received, and (c) enacted according to theory/as per protocol.
2. To examine whether the level of intervention intensity/mode of delivery (study arm) is associated with (a) fidelity and (b) the perceived value of the intervention.
3. To examine the extent to which fidelity delivery, receipt, and enactment explain variance in improvements in INFORM’s primary outcome: care aide involvement in formal team communications about resident care.

### The INFORM study

At least 60 to 80% of the nursing home workforce in the USA [22], Canada [23], and England [24] is made up of care aides (also called care assistants, support workers, or nursing assistants) who provide the vast majority of direct care in these settings [25]. Despite close contact with residents and intimate knowledge of residents’ care needs and preferences [26], care aides remain a largely unregulated workforce with low levels of education and wages [25]. They are rarely involved in decision making about resident care [27]. Care decisions tend to be the purview of regulated staff, and top-down decision making is the norm. However, strong communication is a hallmark of high-quality care, and communication failures are the single biggest contributor to sentinel events [28]. INFORM is a large cluster-randomized trial designed to increase care aide involvement in formal team communications about resident care. The intervention was directed to care unit managerial teams: care managers, the director of care, and those who assist them (e.g., clinical educators)

INFORM has two core components, which are based on goal setting [29] and social interaction theories. (1) *Goal setting activities*: setting specific attainable performance goals that respond to a perceived need to improve care aide involvement in formal team communications about resident care, specifying strategies for goal attainment, and measuring goal progress (the feedback element in goal-setting theory). (2) *Opportunities for participating teams to interact* throughout the intervention to share progress and challenges and learn effective strategies from one another. In early 2016, baseline data on care aide involvement in formal team communications about resident care and other measures of context was collected and fed back, using oral presentations and a written report, to 201 unit teams in 67 Western Canadian nursing homes. To avoid contamination, homes rather than unit teams were randomized to one of three INFORM study arms: *simple feedback* (control) that included only the oral and written dissemination already delivered, *basic-assisted feedback*, and *enhanced assisted feedback*. Managers on units in the *basic* and *enhanced-assisted feedback* arms were invited to attend three workshops over a 10-month period (June 2016–April 2017) and were encouraged to bring 1–3 other unit members who they deemed appropriate for working on increasing care aide involvement in formal care communications (e.g., educational specialists or Directors of Care who work across units in a facility, care aides, nurses). Workshops included a variety of activities to help with goal setting and goal attainment, support from facilitators, progress reporting by participating teams at workshops 2 and 3, and inter-unit networking opportunities.

In the enhanced-assisted feedback arm, all three workshops were face to face. In the basic-assisted feedback arm, the first workshop was face to face and the second and third workshops were conducted virtually using webinar technology (virtual workshops were 1.5 h—half the length of the face to face workshops). The main trial results showed a statistically significant increase in care aides’ involvement in formal team communications about resident care in both the basic and enhanced assisted feedback arms compared to the Simple Feedback arm. However, no differences were observed between the basic and enhanced-assisted feedback arms [12].

## Methods

We conducted a mixed methods, concurrent process evaluation [5, 10] during the INFORM trial to assess intervention fidelity, and experiences of participant teams. During all three intervention workshops, we collected data using attendance lists, intervention delivery checklists, participant team worksheets, exit surveys, and expert observations.

### Data collection

During the first workshop, teams completed a goal setting worksheet. They outlined their specific INFORM goal to increase care aide involvement in formal team communications about resident care, strategies for goal attainment, and measures to provide feedback to teams on goal progress. At the second and third workshops, each team made a presentation about their activities and goal progress since the previous workshop. Study investigators with expertise in the core components of INFORM carried out structured observations of the presentations. At the end of each workshop, teams also completed an exit survey, and workshop facilitators completed an intervention delivery checklist indicating whether each workshop agenda item was delivered as planned.

### Sample

This study includes 106 nursing home care units randomized to basic and enhanced-assisted feedback arms. These 106 units are clustered in 33 different nursing homes (range of 1–10 units per home, median = 3).

### Measures

*Intervention fidelity* is measured using 11 items (Table 1) that reflect fidelity delivery (4 items), receipt (4 items), and enactment (3 items). All 11 items show sufficient variation. Three authors (LG, MH, PN) reached a consensus that these items reflect delivery, receipt, and enactment of the core components of INFORM described above. Because these items reflect different aspects of fidelity rather than a single fidelity construct, they are not scaled together.

**Table 1:** Intervention fidelity items

**Data source**	**Fidelity DELIVERY items**
Workshop attendance records	Number of workshops attended: 0–3
	Continuity of representation at workshops (at least one team member attended more than one workshop): Y/N binary item
	Interteam activities delivered to team at workshop 2 (more than one facility participated in their workshop): Y/N binary variable
	Interteam activities delivered to team at workshop 3 (more than one facility participated in their workshop): Y/N binary variable
	**Fidelity RECEIPT items**
Workshop 1 exit survey	Goal setting workshop content was relevant to my day-to-day work: 5-point agreement Likert scale
Goal setting worksheet	Expert assessment of whether team defined a challenging but attainable, specific, measurable goal: Y/N binary variable
	Expert assessment of whether team defined strategies for goal attainment: Y/N binary variable
	Expert assessment of whether team defined measures for tracking goal progress: Y/N binary variable
	**Fidelity ENACTMENT items**
Workshop 1 exit survey	Completed preworkshop 1 exercise: Y/N binary variable
Workshop 2 observer rating	Team measured impact of changes designed to improve formal team communications: Y/N binary variable
Workshop 3 exit survey	Unit manager time spent planning INFORM activities: 1 ≤ 1 h/week, 2 = 1–2 h/week, 3 = 3+ h/week on average
Workshop facilitators (single-item enactment rating completed post workshop 3)	Overall fidelity enactment: 1–5; 1 = very low enactment, with no/almost no activities undertaken to improve care aide involvement in formal team communications about resident care; 5 = very high enactment, with extensive activities undertaken

*Perceived value of the intervention*. Four measures of team perception of intervention value are based on exit survey data from the three workshops. These measures include team perceptions for (1) the value of workshop 1 material (average of 6 items; e.g., *The preworkshop exercise was valuable, the presentation on SMART goals was valuable*, alpha = .89), (2) the value of workshop 1 inter-team activities (average of 2 items; e.g., *Discussions/feedback from other teams helped with setting performance goals*, alpha = .64)^2^, (3) the value of workshop 2 (average of 3 items; e.g., *Creating the report back presentation was valuable*, alpha = .86), and (4) the value of workshop 3 (average of 3 items; e.g., *Discussion period after report back was valuable*, alpha = .81). All items used a 5-point agreement Likert scale.

*Overall fidelity enactment* reflects an expert assessment of a team’s implementation of the core intervention components in the intended situation. We measured it with a single-item enactment rating scale (1 = very low enactment, with no/almost no activities undertaken to improve care aide involvement in formal team communications about resident care; 5 = very high enactment, with extensive activities undertaken). The rating was provided at the end of workshop 3 jointly by the two raters who delivered all three INFORM workshops and who were most familiar with each team’s activities. These raters observed team progress presentations and had many informal conversations with teams during the workshops. While the raters had limited or no contact with teams between workshops, this kind of global rating scale has been shown to provide a faithful reflection of competency when completed by subject-matter experts in the context of time-limited interactions during Objective Structured Clinical Exams [30, 31]. Fidelity enactment is a binary variable, generated by recoding 1–3 as *lower enactment* and 4 and 5 as *higher enactment*.

*Outcome: Care aide involvement in formal team communications about resident care* is one of 10 concepts measured by the Alberta Context Tool, a comprehensively validated tool to assess modifiable features of the care unit work environment [32]. We used a modified score for formal team communications, asking care aides how often (in the last typical month) they participated in (a) team meetings about residents, (b) family conferences, and (c) change-of-shift reports (each item rated from 1 = never to 5 = almost always). The modified score was generated by recording each item (1 and 2 to 0, 3 to 0.5, 4 and 5 to 1) and summing recoded values (possible range: 0–3). To gather data on formal team communications, we administered the Alberta Context Tool by computer-assisted structured personal interview to a minimum of 10 care aides on each unit participating in the INFORM trial, at baseline (2 months before INFORM) and follow-up (2 months after the last support workshop).

#### Analysis

For study objective 1, we used descriptive statistics to examine the fidelity with which the INFORM intervention was delivered, received, and enacted. For study objective 2, we used chi-square and Fisher’s exact tests to examine whether intervention intensity/mode of delivery (study arm) was associated with differences in fidelity. A Shapiro-Wilk test showed the *Perceived Intervention Value* variables to be non-normally distributed (*p = .000* for all four variables). The Mann-Whitney *U* test was therefore used to examine whether the perceived value of the intervention workshops differed by study arm. For study objective 3, we used hierarchical mixed modeling (GLMM ML estimation, SAS), which accounts for the clustering of units within facilities. This modeling examines the variance in INFORM’s primary outcome (care aide involvement in formal team communications about resident care) that is explained by each of the 11 fidelity delivery, receipt, and enactment items. The posttest score was the dependent variable with the baseline score entered as a covariate. Lastly, we conducted repeated measures analysis of variance to examine whether the relationship between time (baseline and follow-up) and care aide involvement in formal team communications about resident care was moderated by overall fidelity enactment. In other words, did improvement in care aide involvement over the study period differ for low- and high-enactment teams? We did not include a random facility-level intercept in our repeated measures model because our hierarchical mixed modeling results (objective 3) suggested that the variance explained by facility clustering was small and statistically non-significant (facility-level random intercept = 0.0002, *p =* 0.3733, intra-cluster correlation = 0.0411).

## Results

### Fidelity delivery (Table 2)

Fourteen percent of units (15/106) did not participate in any workshop while 63% of units participated in all three workshops.^3^ There were no statistically significant differences by study arm (chi-square = 3.44, df = 3, *p =* .33). Of the 87 units that participated in more than one workshop, 79% had continuity of representation at workshops (the same unit representative attended more than 1 workshop), with no statistically significant differences by study arm (*p =* .41, Fisher’s exact test). At the second workshop, inter-team activities were delivered to all 34 teams in the enhanced-assisted feedback arm that attended but to only 69% of teams in the basic-assisted feedback arm (*p =* .000, Fisher’s exact test). At the third workshop, inter-team activities were delivered to 89% of enhanced-assisted feedback arm teams but to only 72% of teams in the basic-assisted feedback arm (not significant).

**Table 2:** Intervention fidelity and experience descriptives

Fidelity DELIVERY	All units, % (***N***)	Study arm: basic assisted feedback, % (***N***)	Study arm: enhanced-assisted feedback, % (***N***)	Significance
Number of workshops attended				
0	14.2 (15/106)	14.8 (9/61)	13.3 (6/45)	*p = .33*^*a*^
1	3.8 (4/106)	6.6 (4/61)	0 (0/45)	
2	18.9 (20/106)	19.7 (12/61)	17.8 (8/45)	
3	63.2 (67/106)	59 (36/61)	68.9 (31/45)	
Continuity of representation at workshops—% yes	79.3 (69/87)	81.3 (39/48)	76.9 (30/39)	*p = .41*^b^
Interteam activities delivered (workshop 2)—% yes	81.7 (67/82)	68.8 (33/48)	100 (34/34)	*p = .00*^b^
Interteam activities delivered (workshop 3)—% yes	80.6 (58/72)	72.2 (26/36)	88.9 (32/36)	*p = .07*^b^
**Fidelity RECEIPT**				
Relevance of first workshop content				
% *agree*	30.8 (28/91)	38.5 (20/52)	20.5 (8/39)	*p = .06*^b^
% *strongly agree*	69.2 (63/91)	61.5 (32/52)	79.5 (31/39)	
% of teams that defined an appropriate goal at close of workshop 1	92.5 (74/80)	98 (48/49)	83.9 (26/31)	*p = .03*^b^
% of teams that defined strategies for goal attainment at close of workshop 1	97.5 (78/80)	100 (49/49)	93.5 (29/31)	*p = .15*^b^
% of teams that defined measures for tracking goal progress at close of workshop 1	85 (68/80)	89.8 (44/49)	77.4 (24/31)	*p = .12*^b^
**Fidelity ENACTMENT**				
% of teams that completed preworkshop 1 exercise	98.9 (90/91)	100 (52/52)	97.4 (38/39)	*p = .43*^b^
% of teams that measured impact of changes designed to improve formal team communications at workshop 2	67.1 (55/82)	66.7 (32/48)	67.6 (23/34)	*p = .56*^b^
Average h/week manager spent planning INFORM activities:				
< 1	39.7 (25/63)	61.8 (21/34)	13.8 (4/29)	*p = .00*^*a*^
1–2	44.4 (28/63)	35.3 (12/34)	55.2 (16/29)	
3+	15.9 (10/63)	2.9 (1/34)	31.0 (9/29)	
% of teams rated as *higher enactment* at workshop 3	61.4 (44/72)	55.6 (20/36)	66.7 (24/36)	*p = .23*^b^
**Perceived intervention value**	**Mean (SD)**	**Mean (SD)**	**Mean (SD)**	
Value of workshop 1 material	4.43 (0.50)	4.36 (0.51)	4.51 (0.48)	*p* = .132^c^
Value of workshop 1 interteam/researcher interactions	4.49 (0.53)	4.39 (0.59)	4.63 (0.43)	*p* = .069^c^
Value of workshop 2	4.53 (0.48)	4.33 (0.47)	4.78 (0.35)	*p* = .000^c^
Value of workshop 3	4.52 (0.48)	4.25 (0.46)	4.78 (0.34)	*p* = .000^c^

^a^Chi-square test; ^b^Fisher’s exact test; ^c^Mann-Whitney *U* test

### Fidelity receipt (Table 2)

All 91 teams that attended the first workshop *agreed* or *strongly agreed* that workshop content was relevant to their day-to-day work. A higher proportion of teams in the enhanced-assisted feedback arm *strongly agreed* compared to teams in the basic-assisted feedback arm (80% versus 62%, *p = .05*, Fisher’s exact test). Of 80 teams that submitted a goal setting worksheet at the end of workshop 1, expert assessment of fidelity receipt was high: 93% of teams defined an appropriate goal, 98% defined strategies for goal attainment, and 85% defined measures to track goal progress. Only goal definition differed significantly by study arm: 84% of teams in the enhanced-assisted feedback arm defined appropriate goals compared to 98% of teams in the basic-assisted feedback arm (*p =* .03, Fisher’s exact test).

### Fidelity enactment (Table 2)

Nearly all teams in both study arms (90/91) completed the brief preworkshop 1 exercise that was sent to all managers. At workshop 2, 67% of teams in both study arms had measured the impact of changes put in place to increase care aide involvement in formal team communications about resident care. At workshop 3, managers in the basic-assisted feedback arm reported spending fewer hours per week on INFORM-related activities (62% spent < 1 h/week, 2% spent 3+ h/week) than managers in the enhanced-assisted feedback arm (14% spent < 1 h/week, 31% spent 3+ h/week, chi-square (2, *N* = 63) = 18.3, *p = .000*).

### Perceived value of the intervention (Table 2)

Results of the Mann-Whitney *U* test show that teams found workshop 1 to be valuable, with no significant difference between study arms (Table 2). While teams in both arms rated the value of the second and third workshops highly, teams in the enhanced-assisted feedback arm had significantly higher scores than teams in the basic-assisted feedback arm for workshop 2 (4.8 versus 4.3, *U* = 374, *p* = .000) and for workshop 3 (4.8 versus 4.3, *U* = 228, *p* = .000) with large effect sizes (approximately 1 standard deviation).

Hierarchical mixed model results show that, after controlling for pretest formal team communications scores, higher ratings of the relevance of the goal-setting workshop (*strongly agree* vs *agree*) are associated with higher posttest formal team communications scores (*F* = 4.7, *p =* 0.04). The variable reflecting whether *teams measured the impact of changes designed to improve formal team communications between the first two workshops* was also associated with posttest formal team communications scores. However, the fixed effects estimates show that teams who presented on their progress between the first two workshops (whether or not they measured the impact of changes designed to improve formal team communications) had significantly lower posttest formal team communications scores than teams that were not present at the second workshop (*t* = − 2.5, *p* = 0.02 for the group of units that did not measure the impact of changes; *t* = − 2.1, *p* = 0.04 for the group of units that measured the impact of changes). None of the other fidelity delivery, receipt, or enactment items had an effect on posttest formal team communications scores when all items are entered into the same model (Table 3).

**Table 3:** Estimates of fixed effects for the outcome of posttest formal team communications score

Fixed effects^**a**^	Level	Estimate (standard error)	***P***	95% confidence interval
1. Baseline formal team communications score	Continuous	0.24 (0.10)	0.02*	0.03 to 0.44
2. Study arm	Basic-assisted feedback	0.11 (0.07)	0.14	− 0.04 to 0.25
	Enhanced-assisted feedback	Reference	.	
3. Number of workshops attended	2		.	
	3		.	
4. Continuity of representation at workshops	No	0.02 (0.08)	0.76	− 0.14 to 0.19
	Yes	Reference	.	
5. Interteam activities delivered (workshop 2)	No	− 0.09 (0.08)	0.30	− 0.26 to 0.08
	Missing		.	
	Yes	Reference	.	
6. Interteam activities delivered (workshop 3)	No	− 0.01 (0.10)	0.95	− 0.20 to 0.19
	Missing	0.09 (0.16)	0.58	− 0.23 to 0.42
	Yes	Reference	.	
7. Relevance of 1st workshop content:	Agree	− 0.16 (0.08)	0.04*	− 0.31 to − 0.01
	Strongly Agree	Reference	.	
8. Team defined an appropriate goal at close of workshop 1	No	0.18 (0.14)	0.21	− 0.10 to 0.46
	Missing	0.03 (0.10)	0.78	− 0.17 to 0.23
	Yes	Reference	.	
9. Team defined strategies for goal attainment at close of workshop 1	No	0.14 (0.23)	0.55	− 0.32 to 0.60
	Missing		.	
	Yes	Reference	.	
10. Team defined measures for tracking goal progress at close of workshop 1	No	− 0.20 (0.15)	0.17	− 0.49 to 0.09
	Missing		.	
	Yes	Reference	.	
11. Team measured impact of changes designed to improve formal team communications at workshop two	No	− 0.34 (0.14)	0.02*	− 0.62 to − 0.07
	Missing	Reference	.	
	Yes	− 0.27 (0.13)	0.04*	− 0.52 to − 0.01
12. Average h/week manager spent planning INFORM activities:	< 1 h per week	− 0.01 (0.10)	0.91	− 0.22 to 0.20
	1–2 h per week	− 0.06 (0.10)	0.52	− 0.26 to 0.13
	Missing	0.01 (0.15)	0.93	− 0.29 to 0.32
	≥ 3 h per week	Reference	.	

^a^The variable % of teams that completed preworkshop 1 exercise was excluded from the mixed model due to very low variance—see Table 2

Note: Level = “Missing” refers to the group of teams that were absent from the workshop at which those variables were measured

*Significant, *P*<0.05

Absence from a workshop leads to identical missing patterns for variables measured at that workshop. As a result, missing cases are completely collinear for two bundles of variables in Table 3 (8, 9, and 10; 5 and 11) and between variables 3, 6, and 11. Parameters could therefore not be estimated for the missing groups for variables 3, 5, 9, and 10. Variables in each bundle remain independent, and we have therefore retained all of them in the mixed model [33, 34].

**Fig. 1:**
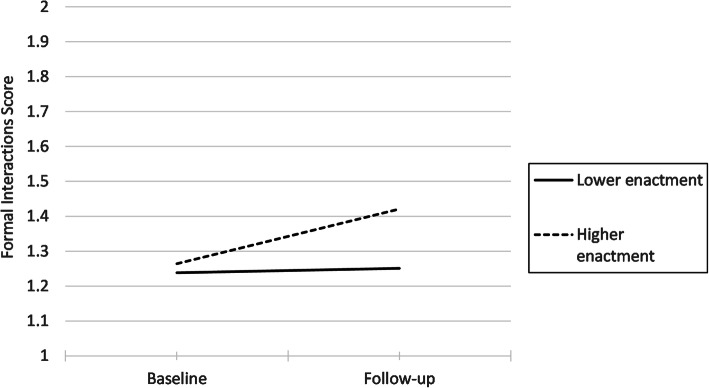
Interaction between time and study arm

Mixed ANOVA (GLM repeated measures) was used to determine the effect of overall fidelity enactment (*low* or *high*) and *time* (baseline to follow-up) on formal team communications (INFORM’s primary outcome). The interaction between *time* and *degree of enactment* is significant (*F*(1, 70) = 4.27, *p* = .042), indicating that improvement in formal team communications between baseline and follow-up differed for *low* and *high enactment* teams. *High enactment* teams showed a larger improvement (increased by more than ½ a standard deviation from 1.25 at baseline to 1.42 at follow-up) (Fig. 1).

## Discussion

We found that the fidelity of the INFORM intervention was moderate to high. Core components of INFORM were successfully *delivered* to most units. Fidelity *receipt* was reasonably high, with > 85% of teams defining (1) appropriate goals to improve care aide involvement in formal team communications, (2) strategies for goal attainment, and (3) measures to give feedback to teams on goal attainment. Data on key markers suggest high levels of fidelity *enactment* at intervention start and moderate enactment at workshops 2 and 3. Study arms had few differences in the extent of fidelity delivery, receipt, and enactment. This helps us understand INFORM trial results: basic- and enhanced-assisted feedback arms had significantly higher follow-up team communication scores than the simple feedback arm, but the two assisted feedback arms did not differ in scores [12].

We examined whether variance in follow-up scores for formal team communications was explained by fidelity delivery, receipt, and enactment items (entering all 11 variables in the same model). Few individual fidelity elements were significantly associated with our main study outcome (care aide involvement in formal team communications about resident care), after controlling for differences in baseline scores. Despite low variance in perceived relevance of the initial goal-setting workshop, units with the highest perceived relevance ratings had higher formal team communications scores at follow-up. Most important are repeated measures results showing that *high enactment* teams saw a significantly larger improvement in formal team communications between baseline and follow-up than *lower enactment* teams (Fig. 1).

Perhaps most novel are our results that overall fidelity enactment is associated with improvements in formal team communications, but the study arm is not. This suggests that intervention intensity/mode of delivery is less important than the intensity with which intervention participants *enact the core components of an intervention*. Best outcomes may come from scaling back the intensity of delivering complex behavioral interventions, instead using scarce resources to support fidelity enactment (i.e., helping teams to successfully implement an intervention). Ways to strengthen enactment may also achieve longer-term sustainment of practice changes in an intervention. We encourage further research on the enactment-sustainment relationship, given that sustainability continues to be a key translational research problem [35, 36].

### Study strengths and weaknesses

Fidelity measures tend to be intervention-specific and may lack rigorous psychometric testing [17, 37, 38]. A strength of our study is multiple data collection methods to assess fidelity, including the gold standard—observation [9]. We established the content validity of items using theory and expert agreement. We found a relationship between fidelity enactment and intervention outcomes that supports the predictive validity of our overall enactment score.

The need to “balance standardization of [intervention] form and content with responsivity to context” [20] encapsulates the fidelity-adaptation debate. We needed to be lenient in assessing aspects of fidelity in teams, which reduced variation on some fidelity items. For example, 92.5% of teams were judged to have defined an appropriate goal at the close of workshop 1, and 97.5% had defined strategies for goal attainment. This may reduce the explanatory power of these fidelity receipt variables in our hierarchical mixed model. Our mixed model may also have low statistical power.

This paper assesses fidelity quantitatively, although qualitative approaches can give a greater depth of understanding and reveal important aspects of complex organizational environments for interventions. We conducted focus groups only across teams, preventing analysis at the team level.

### Contributions to the fidelity and implementation literature

Assessing fidelity is key to understanding care delivery interventions, revealing how and why interventions succeed or fail [5, 39]. However, most trials do not report comprehensive fidelity assessments [9, 17, 38, 40]. This study assesses fidelity delivery, receipt, and enactment, responding to calls for fidelity substudies in audit and feedback trials specifically [41] and to calls for robust, comprehensive, and quantitative evaluations of fidelity in intervention studies more generally [7, 40]. This study also responds to broader calls for theory-based, concurrent process evaluations of complex trials [10], amidst a landscape of process evaluation work that is mainly retrospective and often not theory-guided [42].

Our concurrent fidelity analysis helps us interpret the main results of the INFORM trial. Our results enhance understanding of impact mechanisms in complex trials. Our findings raise questions about the relative importance of intervention intensity and intensity with which participants *enact the core components of the intervention*. The fidelity-outcome relationship has been examined in only a few settings [21], and the results are inconsistent. A systematic review of health promotion and prevention programs found that level of implementation fidelity affects outcomes [21], but a systematic review of psychotherapy outcomes in youth found only a very modest link between fidelity and outcomes [43]. The first review examines fidelity with a strict construct definition, while the second does not explore aspects of fidelity enactment. Our results fill knowledge gaps [44] in how specific aspects of fidelity such as delivery, receipt, and enactment contribute to intervention outcomes, but knowledge gaps regarding fidelity assessment in complex trials remain and require further exploration.

## Conclusions

This concurrent fidelity evaluation demonstrates (1) implementation of the INFORM trial largely as intended, with few differences across study arms and (2) lower levels of fidelity enactment than fidelity delivery and receipt across study arms. Our evaluation highlights the relationship between fidelity enactment and intervention outcomes, and the need for additional research on how best to support intervention enactment. Our findings help explain the main INFORM trial results, strengthening conclusions on INFORM’s effectiveness, and helping to make clearer its mechanisms of impact. This is valuable for replication. Future work on fidelity assessment would ideally combine quantitative and qualitative approaches for both breadth and depth of understanding on ways that interventions are delivered, received, and enacted. Fidelity to core components of interventions is important, but further research must answer precise questions about how, when, and what type of intervention adaptation can positively influence trial effectiveness. Greater attention to fidelity assessment, fidelity measurement, and publication of fidelity results through studies such as this one is needed in the literature to improve the utility of published trials.

## Data Availability

The data used for this article are housed in the secure and confidential Health Research Data Repository (HRDR) in the Faculty of Nursing at the University of Alberta (https://www.ualberta.ca/nursing/research/supports-and-services/hrdr), in accordance with the health privacy legislation of participating TREC jurisdictions. These health privacy legislations and the ethics approvals covering TREC data do not allow public sharing or removal of completely disaggregated data from the HRDR, even if de-identified. The data were provided under specific data sharing agreements only for approved use by TREC within the HRDR. Where necessary, access to the HRDR to review the original source data may be granted to those who meet pre-specified criteria for confidential access, available at request from the TREC data unit manager (https://trecresearch.ca/about/people), with the consent of the original data providers and the required privacy and ethical review bodies. Statistical and anonymous aggregate data, the full dataset creation plan, and underlying analytic code associated with this paper are available from the authors upon request, understanding that the programs may rely on coding templates or macros that are unique to TREC.

## Abbreviations

INFORM: Improving Nursing Home Care Through Feedback On perfoRMance data
TREC: Translating Research in Elder Care

## Footnotes

1 Most authors have adopted this definition of fidelity from Dusenbury et al. (2003). Other terms used for fidelity are intervention/treatment fidelity, implementation fidelity (or fidelity of implementation—FOI). We use the term fidelity.

2 8-items used to measure the value of workshop 1 were factor analyzed (EFA with oblimin rotation) and loaded on these two factors: value of the *workshop material* and value of the *inter-team activities*. The value of workshops 2 and 3 was assessed using three items each so were not factor analyzed.

3 The mean number of participants representing a unit was 2.6 at the 1st workshop (SD 1.3, range 1–5), 2.0 at the second workshop (SD 1.3, range 1–5), and 1.8 at the 3rd workshop (SD 1.2, range 1–4). 29% of workshop participants were facility-level managers, 31% were unit-level managers, 22% were regulated care staff, 4% were care aides, and 12% other (clinical educators, regional leaders). These proportions were consistent across all three workshops.
